# GLI1+ perivascular, renal, progenitor cells: The likely source of spontaneous neoplasia that created the AGMK1-9T7 cell line

**DOI:** 10.1371/journal.pone.0293406

**Published:** 2023-12-07

**Authors:** Andrew M. Lewis, Gideon Foseh, Wei Tu, Keith Peden, Adovi Akue, Mark KuKuruga, Daniel Rotroff, Gladys Lewis, Ilya Mazo, Steven R. Bauer

**Affiliations:** 1 Laboratory of DNA Viruses, Division of Viral Products, Office of Vaccines Research and Review, Center for Biologics Evaluation and Research, Food and Drug Administration, Silver Spring, Maryland, United States of America; 2 Flow Cytometry Unit, OMPT, Center for Biologics Evaluation and Research, OVRR, Food and Drug Administration, Silver Spring, Maryland, United States of America; 3 Department of Quantitative Health Sciences, Lerner Research Institute, Cleveland Clinic, Cleveland, Ohio, United States of America; 4 TCL and M Associates, Leesburg, Virginia, United States of America; 5 HIVE Team, Office of Biostatistics and Epidemiology, Center for Biologics Evaluation and Research, FDA, Silver Spring, Maryland, United States of America; 6 Division of Cellular and Gene Therapies, Office of Therapeutic Products, Center for Biologics Evaluation and Research, Silver Spring, Maryland, United States of America; National Research Centre, EGYPT

## Abstract

The AGMK1-9T7 cell line has been used to study neoplasia in tissue culture. By passage in cell culture, these cells evolved to become tumorigenic and metastatic in immunodeficient mice at passage 40. Of the 20 x 10^6^ kidney cells originally plated, less than 2% formed the colonies that evolved to create this cell line. These cells could be the progeny of some type of kidney progenitor cells. To characterize these cells, we documented their renal lineage by their expression of PAX-2 and MIOX, detected by indirect immunofluorescence. These cells assessed by flow-cytometry expressed high levels of CD44, CD73, CD105, Sca-1, and GLI1 across all passages tested; these markers have been reported to be expressed by renal progenitor cells. The expression of GLI1 was confirmed by immunofluorescence and western blot analysis. Cells from passages 13 to 23 possessed the ability to differentiate into adipocytes, osteoblasts, and chondrocytes; after passage 23, their ability to form these cell types was lost. These data indicate that the cells that formed the AGMK1-9T7 cell line were GLI1+ perivascular, kidney, progenitor cells.

## Introduction

Studies on the biology of neoplasia in mammalian cells growing in tissue culture began in the early 1940s with the observation that untreated mouse and rat cells passaged in cell culture could develop the capacity to form tumors when inoculated into animals [1, 2]. Since these initial observations, a large body of work has accumulated on this topic. After the observations of Earle and Nettleship [1] and Gey [2], the spontaneous neoplastic development/transformation (SPNDT) that occurred in tissue culture paralleled the neoplasia in animals that was induced by chemical carcinogens [3, 4]. However, the relationship between the tissue-culture model of SPNDT and the animal models was unclear and only recently has been investigated [5]. However, it is interesting to note that a “disappointing uniformity”–based on a variety of shared properties–was recognized as early as the 1960s between neoplastically transformed cell lines that were established from “normal” cells by cell-culture passage and those induced by carcinogens or from naturally occurring tumors [4, 6, 7].

The development and characteristics of the AGMK1-9T7 cell line and the cells established from this cell line at 10-passage (p) intervals from p10 to p40 that represent a tissue-culture model of SPNDT in vitro have been described in two previous reports [5, 8]. The basic characteristics of these cells across the spectrum of neoplasia they represented were described in these reports. These characteristics include: 1) reduced doubling times from 97.6 h at p10 to 18.3 h at p40; 2) mesenchymal to epithelial transition from p35 to p40; 3) the expression at p40 of a tumorigenic/metastatic phenotype detected in three strains of immunodeficient mice (athymic-nude, SCID, CD3 epsilon); 4) patterns/profiles of chromosomal aberrations that evolved from four at p11 (4.7% of the genome) to 247 (27.4% of the genome) at p41; and 5) profiles of miRNA expression across the spectrum of neoplasia from p1/p2, p10, p20, and p40. Based on these data, we hypothesized that the evolution of the phenotypes of the AGMK1-9T7 cells can be interpreted to represent the neoplastic processes of initiation, promotion, and progression.

During the establishment of the AGMK1-9T7 cell line, we observed that only 1–2% of the suspension cells (20 x 10^6^ cells/mL) from the kidney of a female African green monkey kidney (AGMK) formed colonies at days five to seven of culture; these colonies expanded during seven to nine weeks of further culture to create the AGMK1-9T7 cell line. The small number of cells that established this cell line raised questions about the origin of the cells that survived in vitro. Given their ability to survive and divide in cell culture, we considered the possibility that these cells could be the progeny of some type of yet-to-be defined renal stem cell or progenitor cell. In this report, we present data that these surviving cells were GLI1+ perivascular, renal, progenitor cells that in vivo react to renal injury to heal wounds and that were the origin of the AGMK1-9T7 cell line. These GLI1+ cells have been described to “serve as progenitors of interstitial myofibroblasts in renal fibrogenesis” [9, 10].

To compare the AGMK1-9T7 cell line with other AGMK cell lines, we have used the World Health Organization 10–87 VERO cell line, the VERO CCL-81 cell line, the BSC-1 and CV-1 cell lines, and suspension cells from the kidneys of 5 African green monkeys [11–13]. As low-passage VERO cells from passages below p123 (ATCC CCL-81) and World Health Organization 10–87 VERO p132 were not available, the studies in this report were conducted using commercially obtained cell suspensions from the kidneys of five adult (four female and one male) African green monkeys. The suspension cells from the kidney of AGMK1 (African green monkey kidney 1) were used to establish the AGMK1-9T7 cell line and the subpopulations of these cells at 10p intervals [5, 8]. Cells from AGMK1, AGMK2, AGMK3, AGMK4, and AGMK5 were used to determine the efficiency of colony formation when renal cell suspensions were placed in tissue culture. As shown in this report, p13 and p23 cells of the AGMK1-9T7 cell line, as well as p2 and p5 cells from the AGMK4 cell suspension, possessed the ability to differentiate into adipocytes, osteoblasts, and chondrocytes. Furthermore, profiles of miRNA expression by p1 cells from each of these five monkeys were similar, and approximately 70% of the human miRNAs expressed at p1 at significant ‘q<0.05’ levels when compared with the baseline suspension cells have been reported to be associated with human cancers (miRCancer Association Database, October 2019 and May 2020) (See the S1 and S2 Data files in this report).

## Materials and methods

### Cell cultures

#### Development of the AGMK1-9T7 cell line

The details of the establishment and characterization of the AGMK1-9T7 cells have been described in two reports [5, 8]. The first report focused on cell-line development and the association of the potential biomarker miRNAs with the expression of the tumorigenic phenotype expressed by the AGMK1-9T7 p40 –p45 cells [8]. The second report [5] described both the profiles of chromosomal aberrations across the spectrum of neoplasia represented by the six sub-lines established at 10-passage intervals (p1, p2, p11, p22, p31 and p41) during serial passage, and the description of the miRNA profiles expressed in five passages (p1, p2, p10, p20, p40). As described in these reports, AGMK cells used in these studies were obtained from the kidneys of female and male monkeys; they were obtained in suspensions containing 20–30 X 10^6^ cells/mL from Diagnostic Hybrids, Athens, OH (AGMK1 = female, lot No. C-406921; AGMK2 = female, lot No.C-461005, AGMK3 = male, lot No. C-461026), or from Zen-Bio Inc., Research Triangle Park, NC (AGMK4 = female, lot No. X-855092414; AGMK5 = female, lot No. Z-0602100114). Dulbecco’s Modified Eagle’s Medium containing 10% fetal bovine serum (DMEM-10) was used for cell culture. The cells from these five monkeys that grew at p1 were used to examine and compare their profiles of miRNA expression to determine the possible relatedness of the cells from different members of the same species to each other when grown in tissue culture. The S1 Data file—miRNAs expressed in the kidneys of 5 different African green monkeys—in this report shows that the miRNA profiles expressed by each of these 5 monkeys during their first passage (p1) in tissue culture were remarkably similar. Of the miRNAs whose expression was up-regulated, 40 were expressed at log2 fold-change ‘q<0.05’ in the cell suspensions from all 5 monkeys, while 29 were expressed at this level in the cells from 4/5 of these 5 monkeys, and 34 were expressed at this level in the cells from 3/5 of these monkeys. Of the miRNAs whose expression was down-regulated, 13 were expressed in 5/5 at log2 fold-change (q< 0.05); while 49 were expressed in 4/5 of these 5 monkeys, and 56 were expressed at these levels in the cells from 3/5 of these monkeys. (To further examine these data, see SD S2 Data—miRNAs expressed at P1 and their association with cancers in humans—in this report).

To develop the AGMK1-9T7 cell line using AGMK1 suspension cells, a cell bank was prepared at p3 of the suspension cells and cells from this cell bank were grown in tissue culture by seeding three T75 flasks with 9 x 10^5^ cells. These cultured cells were then transferred at 7-day intervals when 95–97% confluent by trypsinization and seeding three additional T75 flasks with 9 x 10^5^ cells/flask and cell banks were prepared at 10-passage intervals. Using the same procedures used to grow the AGMK1-9T7 cells, the cells from AGMK4 were used at either p2, p5, p8 for flow cytometry, indirect immunofluorescence, and differentiation assays.

Because the number of cells in each suspension is limited, we used the suspension cells from the kidneys of 5 different African green monkeys to perform the colony count assays, indirect immunofluorescence assays, western blots, and cell differentiation assays. We initially obtained suspension cells from AGMK1, AGMK2, and AGMK3 and used the cell suspension from AGMK1 to develop the AGMK1-9T7 cell line. The cells from all three monkeys were used to examine the patterns on miRNA expression in these monkeys, and to determine by microscopic counting in 36 different assays the number of colonies produced in culture when the suspension cells from these 3 monkeys were plated out. These counts by microscopic observation revealed that an average of 1.3% of the suspension cells from these 3 monkeys formed the colonies that grew to confluence to establish a cell line. This series of studies exhausted the cell suspensions we had obtained on these 3 monkeys. To continue these studies we obtained suspensions of kidney cells from 2 additional monkeys. The cell suspension from AGMK4 was used to verify the colony counts using another technique, as our agency had just acquired a Celigo Image Cytometer (Nexcelom Bioscience, Lawrence, MA), which could be used to count the total number of colonies on 30-mm petri dishes. The counts obtained with this equipment found that 1.4% to 1.5% of cells were forming colonies when plated out. Counting by microscopic analysis had found that an average of 1.3% of these plated-out AGMK1, AGMK2, and AGMK3 cells formed colonies. In addition to the similarities of colony formation expressed by the kidney cells from these five African green monkeys, our analysis of the patterns of miRNA expression revealed, as outlined above, a close similarity between the patterns of miRNAs expressed at p1 by the kidney cells from these five different monkeys.

The CCl-81 VERO cell line at p123, the BSC-1 cell line at p49, and the CV-1 cell line at p42 were obtained from the ATCC. The 10–87 VERO cell line was passaged by ATCC, and cells at 10-passage intervals (p140 to p250) were cryopreserved [11]. All the cells used in these studies were stored as frozen cell banks in liquid nitrogen and were tested by IDEXX Laboratories, Columbia, MO, for their chromosomal identity (shown to be of AGM origin); they were also shown to be negative for contamination with thirty-three different types of viruses and five species of mycoplasma.

The cells used as negative and positive controls for indirect immunofluorescence were cultures of human skin (Lot No. 62691130 obtained from the ATCC, Manassas, VA) and human kidney (Lot No. 0000364975 obtained from Lonza, Walkersville, MD).

#### Colony counts

To count colony formation by suspensions of African green monkey kidney cells (AGMK1, AGMK2, and AGMK3), these suspension cells were plated out in 60 mm dishes using DMEM-10 at 5x10^4^, 1x 10^5^, 1.5x10^5^ cells per dish and observed for 7–8 days until colonies large enough to be counted were observed. At this stage, the medium was removed, and the colonies were fixed, stained, and observed and counted by microscopic observation.

To develop and count the colonies of the cells that established themselves in culture using the Celigo Image Cytometer, suspensions of AGMK4 cells containing 25,000, 50,000, and 75,000 cells were plated in 30-mm culture dishes (BD Falcon, Franklin Lakes, NJ) in DMEM-10. These cultures were observed daily for seven—eight days, by which time colonies of countable size (three to more than five cells) had formed. When the colonies had attained this size, the medium containing unattached cells was removed and placed in new dishes to check for additional colony formation; observations over a period of four to five weeks revealed that very few new colonies appeared in these dishes. After the cells in the original dishes attached, the medium was removed, the colonies were washed with DPBS, fixed with methanol, and stained with crystal violet (Baxter Health Care Corp, Meekesgan, MI) for 30 min. The number of colonies that developed was determined using a Celigo Image Cytometry Unit, which counts the total number of colonies (three or more cells/colony) on a 30-mm dish.

#### Indirect immunofluorescence for PAX-2, MIOX, CD44, and GLI1

Indirect immunofluorescence assays were used to determine the renal cell origin of the AGMK cells that created the AGMK1-9T7 cell line. PAX-2, MIOX, and GLI1 antibodies have been shown to react with cells from the kidney [14–16]. For indirect immunofluorescence assays, cells were seeded at 1 X 10^4^ cells/mL on 18 mm-coverslips (Neuvitro Corporation, Vancouver, WA) in 12-well dishes, cultured overnight in DMEM-10, and fixed in 4% paraformaldehyde (Alfa Aesar, Ward Hill, MA) for 30 min. Fixed cells were washed 3 times with PBS containing 0.1% Tween 20 (Sigma, St. Louis, MO) in 1X PBS for 5 min/wash. Cells were then blocked with 10% normal goat serum (Millipore, Kankakee, IL) in PBS with 0.1% Triton X-100 (Sigma, St. Louis, MO) for 1 h at room temperature. Cells were incubated overnight at 4°C with rabbit anti-PAX-2 (Cat # GR280334-3 Abcam, Cambridge, MA), rabbit anti-MIOX (Cat # OK17787715, Thermo Scientific, Rockford, IL), anti-CD44 (Cat # A001, Origen, Rockville, MD), and GLI-1 (Cat # sc-5151751 Santa Cruz Biotechnology, Inc Dallas, TX) diluted 1:50 in normal goat serum diluted in 1x PBS containing 0.05% Triton X-100. Next, these cells were washed 3 times (5 min/wash), incubated with Alexa 488-goat anti-rabbit antibody (Cat # A11008 Invitrogen, Eugene, OR) for PAX-2 and MIOX, Alexa Fluor 594 goat anti-mouse antibody (Cat # A11032 Invitrogen, Eugene, OR) for CD44, and Alexa Fluor 488-goat anti-mouse antibody (Cat # A11001 Invitrogen, Eugene, OR) for GLI1 diluted 1:2000 in PBS for 1 h at room temperature with reduced exposure to light. Cells on coverslips were again washed 3 times with reduced exposure to light with PBST for 5 min/wash, and the coverslips were mounted on slides with ProLong^®^ Gold Antifade (Life Technologies, Eugene, OR) with DAPI. The stained cells on the coverslips were examined and pictures were taken using a BZ-9000 BioRevo at 20x magnification (Keyence Corporation of America, Itasca, IL).

#### Western blots

Protein samples were denatured at 95°C for five minutes. They were allowed to cool at room temperature. Next, 30 μg of each protein sample was loaded into wells of a 4–12% SDS-PAGE gel (Invitrogen, Waltham MA; Cat # NPO33BOX). Gels were run for 1–2 h at 100 V and transferred to PVDF membranes using the eBlot system (GenScript, Pistcataway, NJ). Membranes were then blocked at RT for one hour on a shaker with blocking buffer consisting of 5% non-fat dry milk in TBST (TBST is 1%Tween-20 in 150 mM NaCl, 20 mM Tris-HCl, pH7.6). Membranes were washed three times with TBST, five min each. Next, membranes were incubated for one hour in HRP-conjugated Anti-Rabbit lgG secondary antibody (BD Biosciences, Billerica, MA; Cat # 554002) at 1/1000 for the GLI antibody in 5 mL of blocking buffer at room temperature. To verify the presence and level of protein in these reactions this gel was also reacted with β Actin (Cat # 8H10D10, Danvers MA) (See the S3 Data file for the original gel used to develop Fig 7 - in this report). Membranes were washed three times in TBST, five min/wash. They were then incubated in 2 mL of Supersignal West Dura Extended Duration Substrate from Thermo Scientific (Waltham, MA) for five min. For signal development, excess reagent was removed, and membranes were covered in transparent plastic wrap. Images were acquired using a Syngene G Box and Genesys software (Indianapolis, IN).

#### Differentiation assays

To perform the differentiation assays, we used published methods that described using fewer cells per assay and shorter incubation times [17]. For adipogenic assays, cells were seeded at 5 x 10^4^/well in 2 mL of mesenchymal stem cell growth medium (MSC-GM from Gibco, Gaithersburg, MD; Cat # 1257–048) in 12-well plates (Corning). Cells were Incubated at 37°C in a humidified atmosphere with 5% CO_2_. They were fed every 2–3 days by replacing the medium until the cultures became confluent. At 100% confluence, one of the duplicate samples was fed with MSC Adipogenic Differentiation Medium (PromoCell, Heidelberg, Germany; Cat # C-28016), and the second well was fed with MSC-GM as a negative control. Cells were fed every three days by replacing the medium in the first well with fresh MSC Adipogenic Differentiation Medium and with MSC-GM for the negative-control cells in the second well. Cells were observed daily. When small vacuoles had developed in the differentiating cells, the test and negative cultures were fixed and stained. For adipocyte detection, cells were removed from the incubator, washed with Dulbecco’s Phosphate-Buffered Saline (DPBS) without Ca^++^/ Mg^++^. They were fixed with 10% neutral buffered formalin (Alfa Aesar, Ward Hill, MA) at RT for 30 min. They were then incubated in 60% isopropanol for five min and then in diluted Oil Red O (Sigma Life Science, Miamisburg, OH; Cat. # 1320-06-5) for fifteen min. To complete the assay, the cells were covered with DPBS and analyzed for intracellular lipid vesicles, which stained bright red.

For osteogenic assays, suspension cells were plated 3.0 x 10^4^ cells/well in 2 mL of MSC-GM (Gibco; Cat # 1257–048) in 12-well Corning plates. Cells were incubated at 37°C, in a humidified atmosphere with 5% CO_2_. Cells were allowed to reach 100% confluence, which took two days. One of the duplicate samples was fed with MSC Osteogenic Differentiation Medium (PromoCell, Heidelberg, Germany; Cat # C-28013) or with MSC-GM for the second well as a negative control. Cells were fed every three days by replacing the medium with fresh MSC Osteogenic Differentiation Medium or MSC-GM Growth medium for the negative-control well for 28 days. For osteoblasts, the cells were removed from the incubator, washed with DPBS without Ca^++^/ Mg^++^ and fixed with 10% neutral-buffered formalin (Alfa Aesar) at RT for 30 min. Fixed cells were incubated in Alizarin Red S (Sigma Life Science, Miamisburg, OH; Cat # 130-22-3) for 10 min and covered with DPBS for analysis. Undifferentiated cells were colorless, whereas MSC-derived osteoblasts stained bright orange red.

For chondrogenic assays, 5.0 x 10^5^ suspension cells were seeded in 5-mL polypropylene tubes (Eppendorf) to form chondrogenic pellets. Cells were then transferred to a 15-mL polypropylene tube (Sarstedt), washed with incomplete chondrogenic medium (Lonza, Walkersville, MD; Cat # PT-3925), and centrifuged at 150 x g for 5 min at RT; the supernatant was discarded. Cells were resuspended in 1 mL of incomplete chondrogenic medium, centrifuged again at 150 x g for 5 min, and the medium discarded. The cells were resuspended in complete chondrogenic differentiation medium (Lonza; Cat # PT4121) and centrifuged at 150 x g for 5 min at room temperature. Tube caps were loosened one-half turn to allow gas exchange, and the tubes were incubated at 37°C in a humidified atmosphere with 5% CO_2_. Cell pellets were fed with complete chondrogenic differentiation medium every 2–3 days by replacing the medium in each tube. Chondrogenic pellets were harvested after 28 days in culture. Pellets were paraffin embedded and frozen sectioned. Thin sections were then slide-mounted and immune-stained for type II collagen. For chondroblast detection, each slide was placed in 3% acetic acid (Mallinckrodt Inc, Harrisburg, PA; Cat # 64-19-s7) for 3 min followed by incubating in 1% Alcian Blue Stain Solution (Sigma Life Science, Miamisburg, OH; Cat # B8438) for 30 min. The slide was washed in running tap water for 1 min, dipped briefly in ethanol to dehydrate, and air dried. Undifferentiated cells were colorless, whereas MSC-derived chondroblasts stained bright blue.

#### Flow cytometry

For flow-cytometry assays used to detect CD and GLI1 antigens, culture medium from monolayers of cells growing in T150 flask was decanted, the cells were washed at RT with DPBS (without Ca or Mg). Accutase (Innovative Cell Technologies, Inc., Chantilly, VA; Cat # AT104) was added to the cultures, and cells were incubated until they detached. Cell-culture medium was added, and cell clumps were dispersed by gentle pipetting. Cells were then transferred into a 15-mL conical polypropylene tube (Sarstedt), washed with 2–4 volumes of DPBS, centrifuged at 500 g for 5 min, and washed with Flow Cytometry Staining buffer (eBioscience, San Diego, CA; Cat # 00-4222-26). Washed cells were counted using a Cellometer (Nexcelon, Laurence, MA) and were re-suspended to 1X10^7^ cells/mL. One hundred microliters per well containing 1X10^6^ cells were dispensed into a 96-well flat-bottom plate, mixed, and incubated for 30 min at RT in the dark with 10 μL of APC/PE-labeled antibodies CD24, Cat # 12-0247-42; CD29, Cat # 17-0291-82; CD31, Cat # 102410; CD34, Cat # 12-0349-42; CD44, Cat # 12-0441-83; CD45, Cat # 12-0451-82; CD73, Cat # 11-0739-42; CD105, Cat # 12-1057-42; all from eBioscience, San Diego, CA) and Sca-1 (BioLegend, San Diego, CA; Cat # 108126).

For GLI1 flow-cytometry assays, 1X10^6^ cells were dispensed into a 96-well flat-bottom plate, mixed, and incubated for 15 min in fixation and permeabilization solution (BD biosciences San Jose, CA; Cat # 554722). Cells were washed twice and incubated with a primary antibody to GLI1 (ThermoFisher, Waltham, MA; Cat # PA5-72942) at RT for 1 h without exposure to light. Cells were washed twice and incubated with the secondary antibody Alexa Fluor 647 for 30 min (Jackson Immuno Research Labs, West Grove PA; Cat # 711-605-152).

Finally, washed cells that had been incubated with antibodies to all eight CD markers or with antibodies to Sca-1 or GLI1 were re-suspended in 200 μL of staining buffer (eBioscience, San Diego, CA; Cat # 00-4222-26). Data were acquired using BD LSR II Flow Cytometer (Becton Dickinson, BioSciences, San Jose, CA) and BD LSRFortessa^™^ X-20 Flow Cytometer (Biosciences Corp, Piscataway, NJ). Data analysis was performed using FlowJo software (FLOWJO, LLC, Ashland, OR).

## Results

### Cellular origins of the AGMK cells that grow in tissue culture: Colony formation

**Fig 1:**
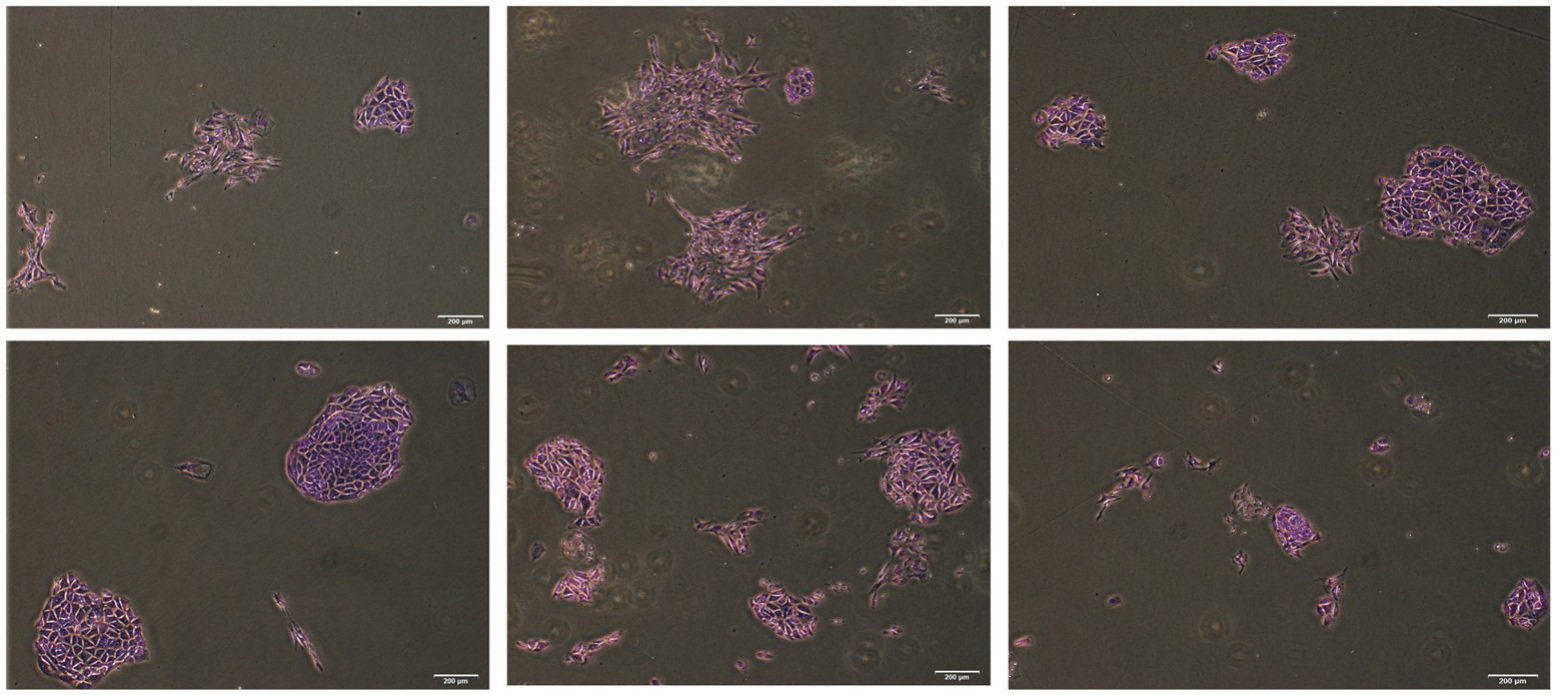
Photograph of colonies that formed by seven days when the suspension cells from AGMK1 were plated in 60-mm dishes. Photographs were taken at 4X magnification using a BZ-9000 BioRevo microscope.

**Fig 2:**
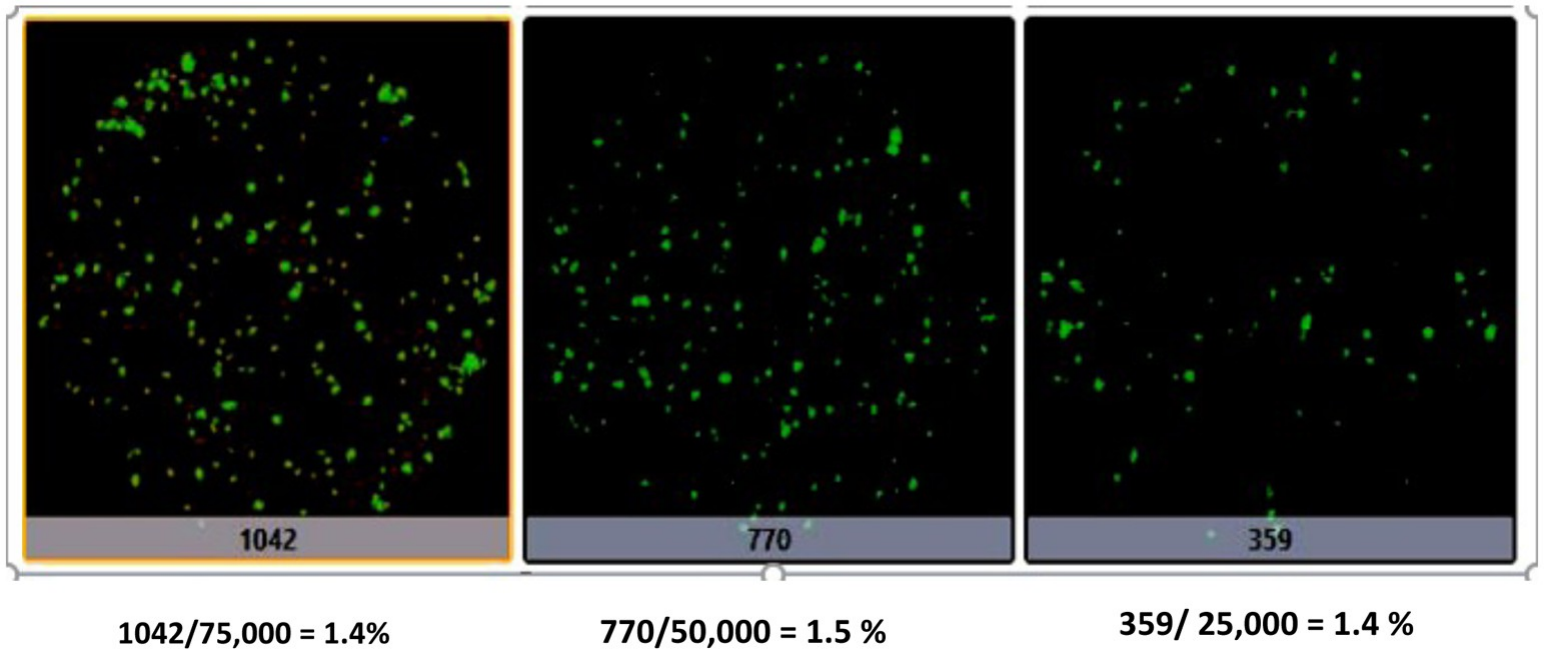
Celigo Image cytometer counts of colonies developed after 3 days in culture from suspensions of AGMK cells plated in 30-mm dishes: % of cells for the three experiments that formed colonies are shown: 1042/75,000 = 1.4%; 770/50,000 = 1.5%; 359/25,000 = 1.4%.

During the establishment of the AGMK1-9T7 cell line, we observed that very few of the cells from the suspension of the AGMK1 formed colonies that grew out to form the initial culture of AGMK cells (Fig 1). Initial counts of the colonies formed by suspension cells from of African green monkey kidneys (AGMK1, AGMK2, and AGMK3) that were done by microscopy had revealed that 1.48% (assay 1), 1.33% (assay 2), and 1.19% (assay 3) of these suspension cells formed colonies in tissue culture after 7–8 days. To verify by a more rigorous technique how many of the suspension cells were forming these colonies, 30-mm dishes were seeded with 25,000, 50,000, and 75,000 viable suspension cells per dish from AGMK4. These assays were repeated twice with these cells and each of these three assays produced the similar results. When these colonies had attained countable size (more than three cells/colony), the medium containing unattached cells was removed and placed in new dishes to check for additional colony formation; very few new colonies appeared in these cultures over more than four weeks. The colonies in the colony-count assays were fixed with methanol at 6–9 days (when they had attained a size that could be readily visualized) and stained with 0.4% crystal violet. To achieve accurate counts, the number of colonies that formed from the AGMK cell suspensions was confirmed in repetitive assays using a Celigo Image Cytometer. Counts of these colonies at p0 in three assays indicated that only 1.4%–1.5% of the suspension cells seeded in culture formed the colonies that were contributing to the AGMK cell monolayer (Fig 2). The cells that grew out resembled mesenchymal cells with a fibroblastic morphology [8]; these cells also resembled the mesenchymal/fibroblasts cells that have been reported to grow in culture from mammalian tissues [1, 18–22].

### Cell lineage

**Fig 3:**
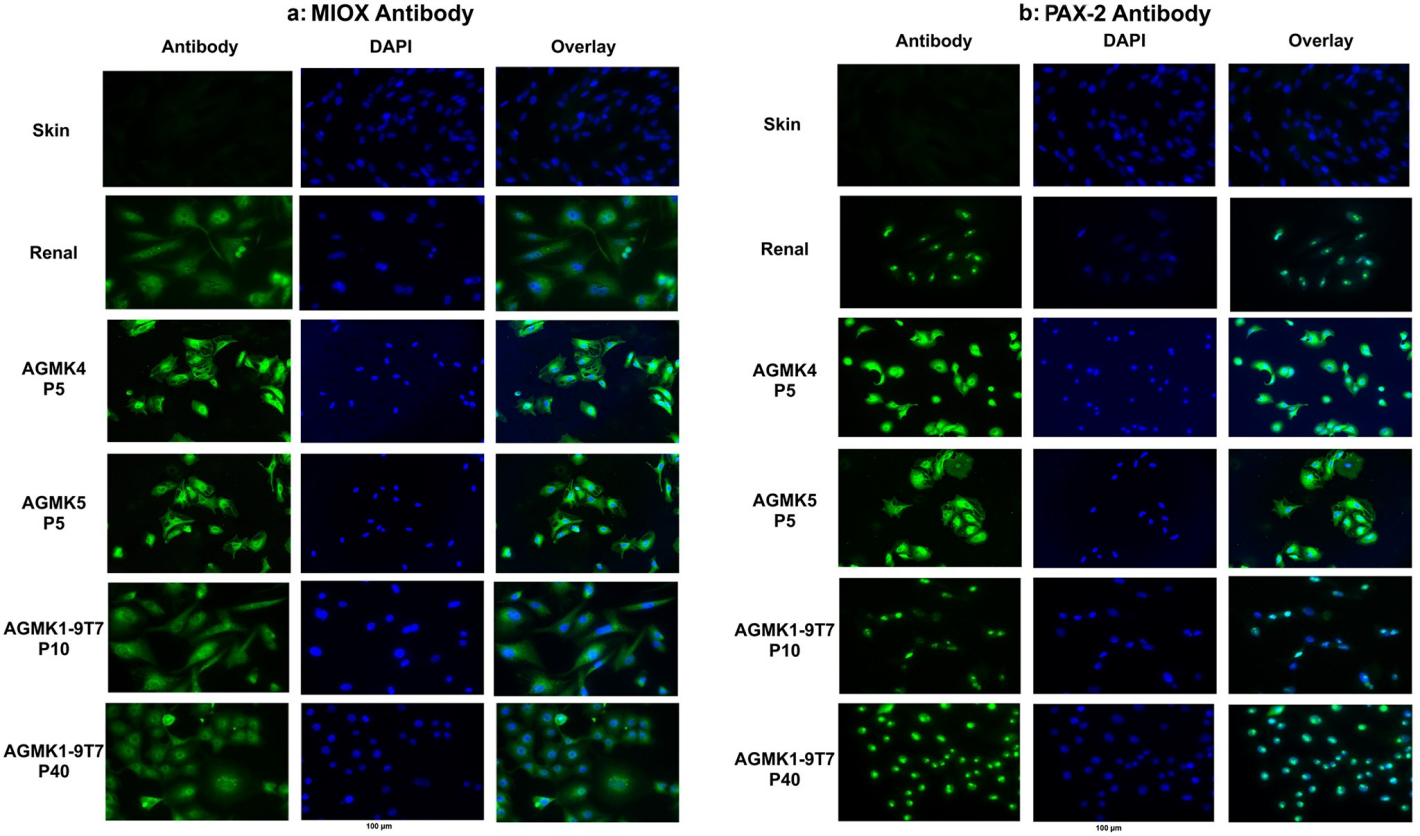
**a** and **b**. Immunofluorescence staining of renal antigens in AGMK4 and AGMK5 cells at p5 and AGMK1-9T7 cells at p10 and p40 using anti-MIOX and anti-PAX-2 antibodies. The human skin and renal cells represent the negative and positive controls for these assays. The cells that react with these antibodies are green. The 100-micrometer scale bar is located under the DAPI reaction with p40 at the base of the figure.

In addition to having cells derived from the kidney, the suspension of cells obtained from the kidney of a monkey will contain cells from other sources, such as from the blood, and some of these cells could be tissue-mature stem cells from the blood. To identify the type of cells that grew out of these suspensions of kidney cells, we needed to identify the lineage of the cells that formed the AGMK1-9T7 cell line. To confirm the renal origin of the cells comprising the AGMK1-9T7 cell line, we tested p10 and p40 cells as well as cells from AGMK4 and AGMK5 for the expression of MIOX and PAX-2 by indirect immunofluorescence (Fig 3). These proteins have been identified as biomarkers for acute kidney injury [16, 23, 24], and both have been shown to be expressed in kidney cells in multiple species, (The Human Protein Atlas; [25, 26]). These data confirmed that the AGMK1-9T7 cell line was derived from renal cells.

### Flow cytometry assays

A number of studies have reported that CD44 and CD105 are expressed by renal cells [27, 28]. To examine the AGMK1-9T7 cell passages for the expression of CD markers, flow cytometry analysis was done using antibodies to 10 proteins (CD24, CD29, CD31, CD34, CD44, CD45, CD73, CD105, Sca-1, and GLI1). These assays were performed repetitively on passages of the AGMK4 cells (p5 and p8) and the AGMK1-9T7 line (p12, p23, p33, p43) (Table 1). These data revealed that fewer AGMK1-9T7 cells reacted with CD29, CD31, CD34, CD45 (reaction levels ranging between 1.3% to 67.3%) but more cells reacted with CD44, CD73, CD105, and Sca-1 (reaction levels ranging between 90.4%s and 99.8%). Similar proportions of cells expressing these proteins have been reported by others for progenitor cells [29].

**Table 1:** Flow-cytometry analysis evaluating the expression of CD markers and Sca-1 by AGMK4 and AGMK1-9T7 cell lines. Human MSC = human mesenchymal stromal cells [30].

Cell Lines	CD Markers																	
	CD24		CD29		CD31		CD34		CD44		CD45		CD73		CD105		Sca-1	
**Reaction level**	**% Neg**	**% Pos**	**% Neg**	**% Pos**	**% Neg**	**% Pos**	**% Neg**	**% Pos**	**% Neg**	**% Pos**	**% Neg**	**% Pos**	**% Neg**	**% Pos**	**% Neg**	**% Pos**	**% Neg**	**% Pos**
**Human MSC**	**97.2**	**2.8**	**42.7**	**58.3**	**69.8**	**30.2**	**95.1**	**4.9**	**0.7**	**99.1**	**96.3**	**3.7**	**2.7**	**97.3**	**0.7**	**99.1**	**1.4**	**98.6**
**293T**	**99.6**	**0.4**	**49.4**	**51.6**	**96.7**	**3.3**	**96.2**	**3.8**	**97.9**	**2.1**	**97.6**	**2.4**	**97**	**3**	**97.9**	**2.1**	**97.6**	**2.4**
**AGMK4 p5**	**85.6**	**14.4**	**33.7**	**67.3**	**96.3**	**3.7**	**88.2**	**11.8**	**0.2**	**99.8**	**98.6**	**1.4**	**7.2**	**92.8**	**0.2**	**99.8**	**3.6**	**96.4**
**AGMK4 p8**	**97**	**3**	**37.8**	**62.2**	**98.2**	**1.8**	**92.4**	**7.6**	**9.6**	**90.4**	**96.9**	**3.1**	**3.5**	**96.5**	**7.4**	**92.6**	**1.8**	**98.2**
**AGMK1-9T7 p12**	**98**	**2**	**42.7**	**58.3**	**40.2**	**59.8**	**96.3**	**3.7**	**3.2**	**96.8**	**98.7**	**1.3**	**3.5**	**96.5**	**3.2**	**96.8**	**2.4**	**97.6**
**AGMK1-9T7 p23**	**97.6**	**2.4**	**47.4**	**52.6**	**44.2**	**55.8**	**98.6**	**1.4**	**2.1**	**97.9**	**95.6**	**4.4**	**1.9**	**98.1**	**2.1**	**97.9**	**3.2**	**96.8**
**AGMK1-9T7 p33**	**97.5**	**2.5**	**43.7**	**57.3**	**37.7**	**62.3**	**94.3**	**5.7**	**3.8**	**96.2**	**96.4**	**3.6**	**4**	**96**	**3.8**	**96.2**	**3.6**	**96.4**
**AGMK1-9T7 p43**	**96.4**	**3.6**	**47.8**	**52.2**	**41.8**	**58.2**	**88.2**	**11.8**	**6.2**	**93.8**	**96.7**	**3.3**	**7.7**	**92.3**	**6.2**	**93.8**	**1.8**	**98.2**

**Fig 4:**
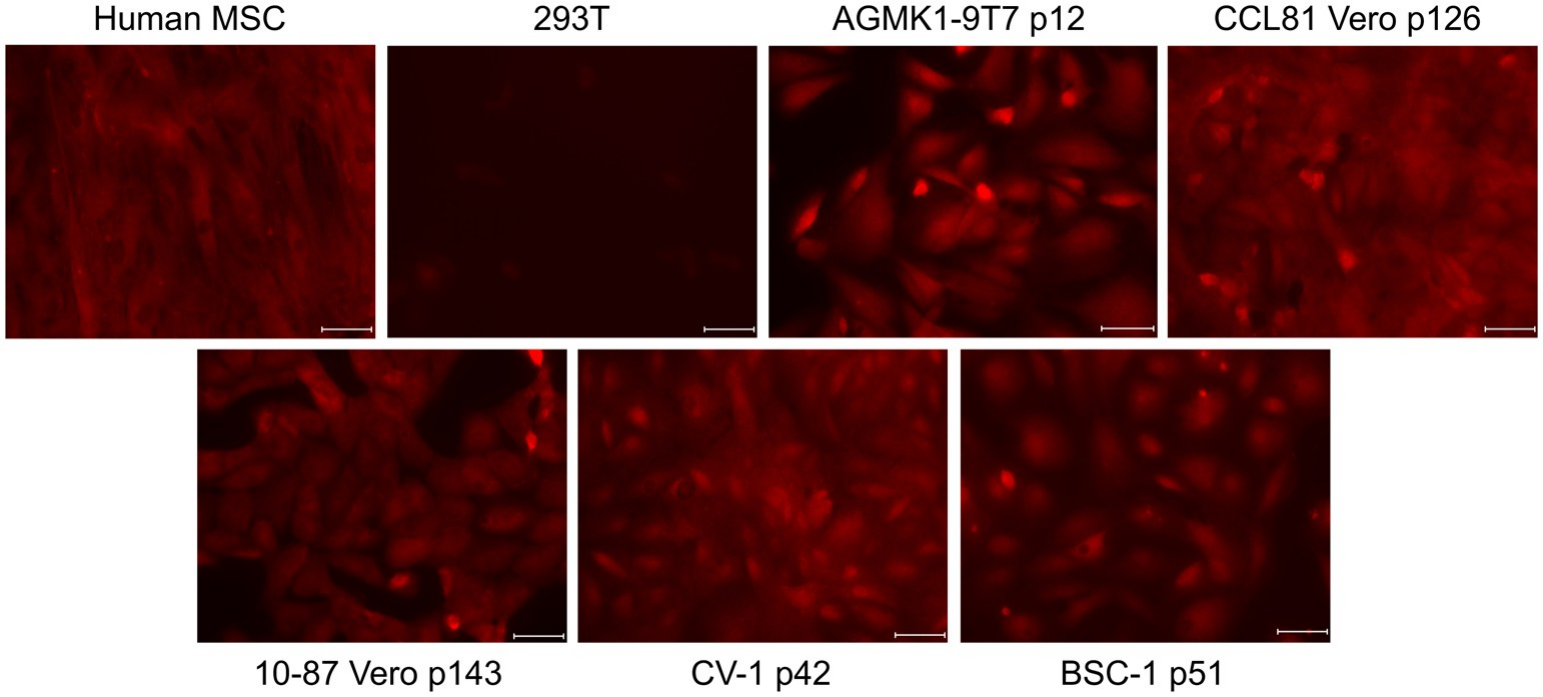
Immunofluorescence (FA) staining of CD44 in AGMK1-9T7 cells at p12 and in other AGMK cell lines. Human MSC = human mesenchymal stromal cells [30]. Photographs were taken at 4X.

These results indicated that 90.4%– 97.9% of the cells in six passages of the AGMK cells expressed the stem/progenitor cell marker CD44. The expression of CD105 by AGMK cells varied from 99.8% at p5 to 93.8% at p43. The reaction of these cells and other AGMK cell lines with antibodies to CD44 was confirmed by indirect immunofluorescence (Fig 4). As shown by others, the expression of both CD44 and CD105 supports the renal origin of these cells [27, 28]. In addition, proportionally fewer AGMK1-9T7 cells (52.2%–67.3%) reacted with CD29, while more AGMK cells (92.3%–98.2%) reacted with antibodies to CD73 and Sca-1.

### Expression of GLI1 by AGMK1-9T7 and other AGMK cell lines

**Fig 5:**
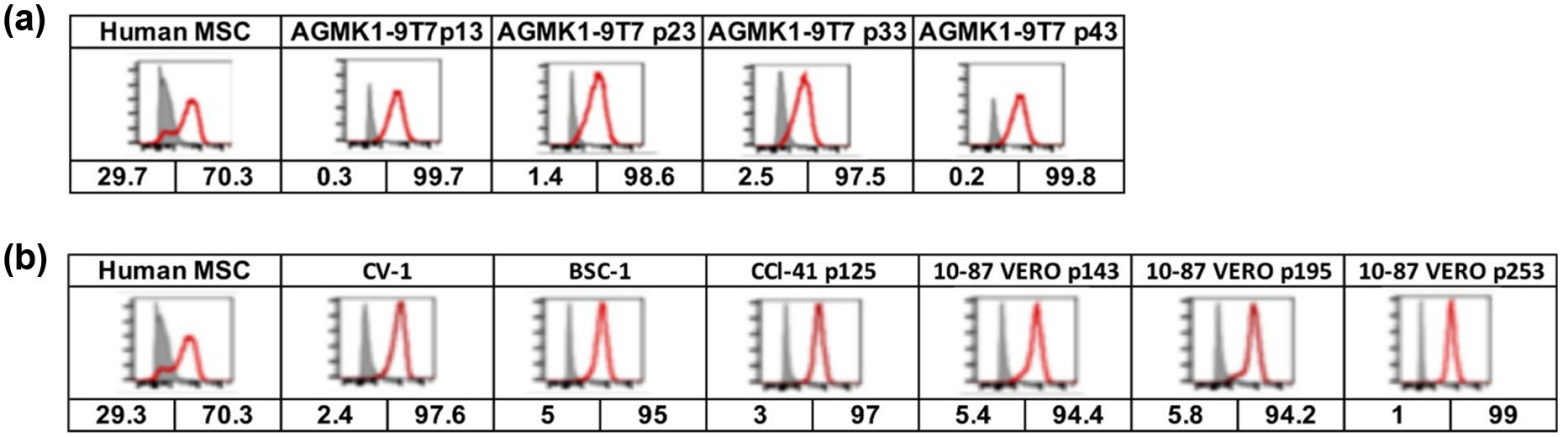
**a**. GLI1 expression in passages of the AGMK4 cells and the AGMK1-9T7 cell lines by flow cytometry. Human MSC = mesenchymal stromal cells [34]. **b**. GLI1 expression in VERO, CV-1, and BSC-1 cell lines by flow cytometry. Human MSC = mesenchymal stromal cells [30].

In 2015, Kramann et al. reported that GLI1+ mesenchymal progenitor cells contributed to organ fibrosis in major organs including the kidney, lung, liver, and heart after they were severely injured [10]. Kramann et al. [10] and Kim and Braun [9] also evaluated the expression of CD29, CD31, CD34, CD44, CD45, CD73, CD105, and Sca-1. As these tissue fibroblasts were reported to be the progeny of mesenchymal progenitor cells that had become unipotent after extensive injury, we considered the possibility that the cells that survived and formed a cell monolayer when placed in culture could represent the progeny of such unipotent mesenchymal progenitor cells. To examine this possibility, we tested the AGMK1-9T7 cell line at different passage by flow cytometry for their expression of the GLI1 protein (Fig 5a). We were surprised to find that all the AGMK cell lines, including VERO, CV-1, and BSC-1, expressed GLI1 (Fig 5b). These data not only support the idea that the cells that formed the AGMK1-9T7 cell line represent the progeny of mesenchymal renal progenitor cells, but also might indicate that this is true for all cell lines derived from the monkey kidney.

**Fig 6:**
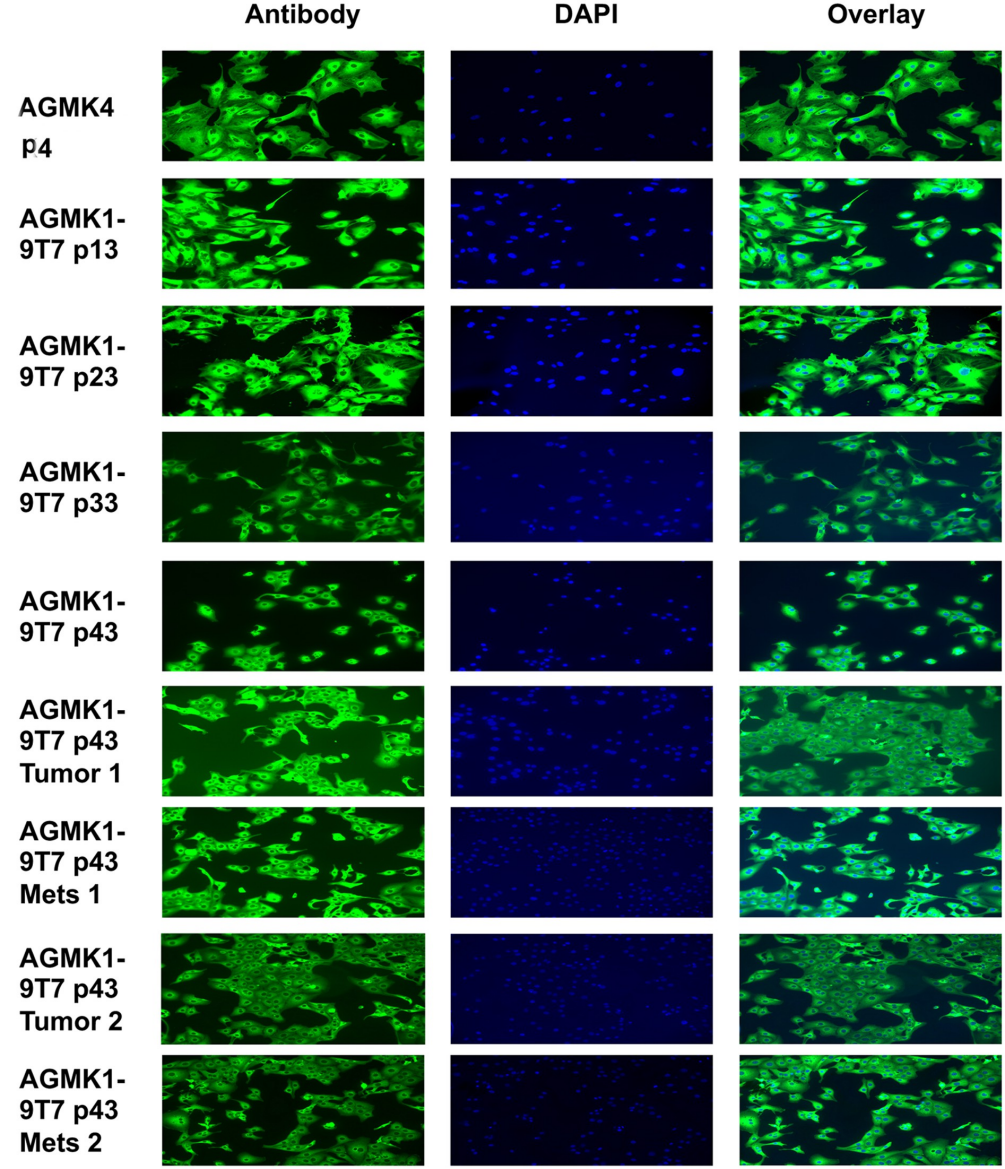
Indirect immunofluorescent assay for GLI1 in AGMK4 p4 cells and different passages of AGMK1-9T7 cells including cells from tumors and their lung metastases. The magnification for these pictures is 4X.

**Fig 7:**
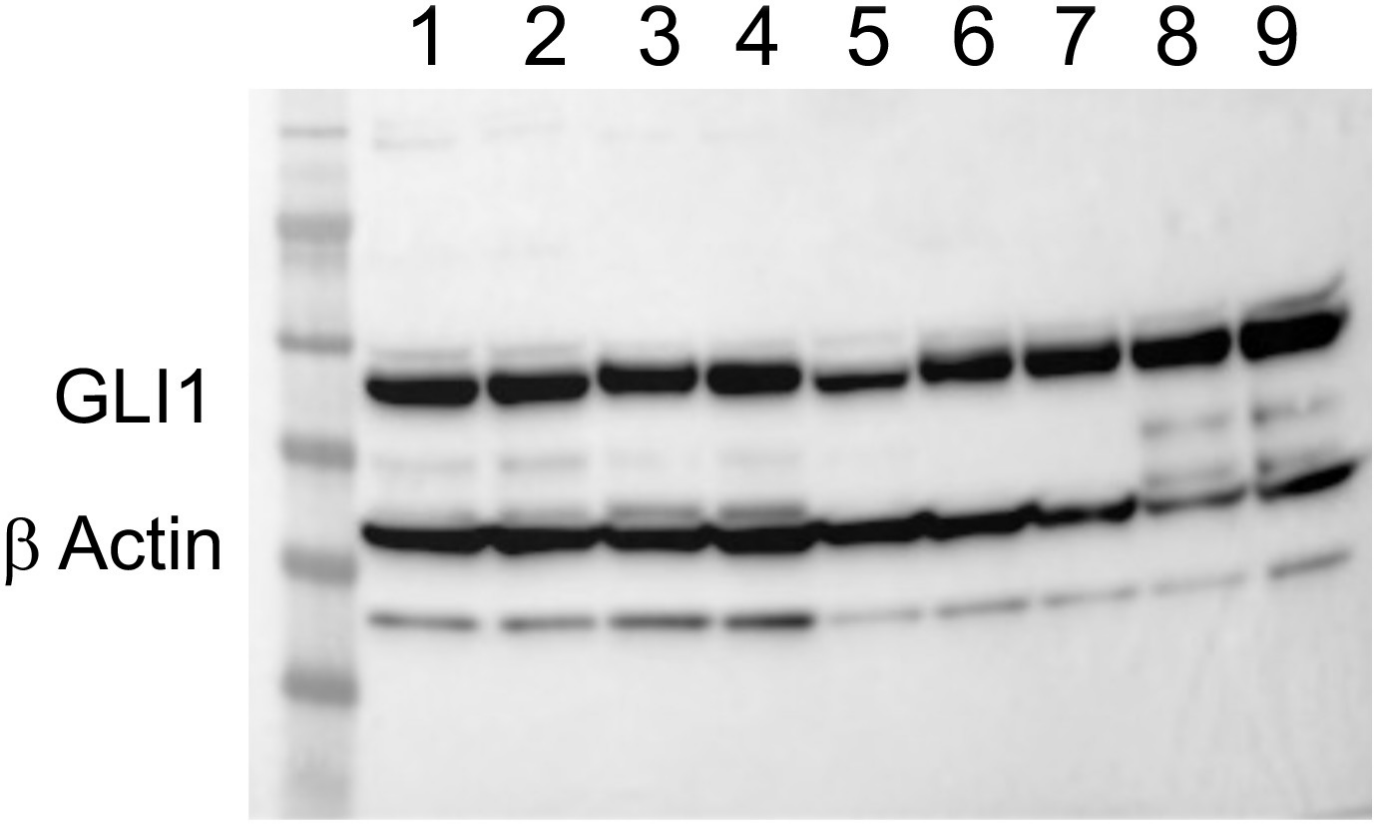
Western blot detecting GLI1 in the AGMK4 and AGMK1-9T7 cells. The β Actin reaction was used to verify the presence and levels of proteins present in this blot. Cell lines tested: 1. AGMK4 p0, 2. AGMK4 p3, 3. AGMK4 p5, 4. AGMK4 p8, 5. AGMK1-9T7 p13, 6. AGMK1-9T7 p23, 7. AGMK1-9T7 p33, 8. AGMK1-9T7 p43, 9. HeLa.

Indirect immunofluorescence (Fig 6) and western blot assays (Fig 7; see S3 Data for the original version of this western blot) were used to evaluate the expression of GLI1 by different passages of the AGMK1-9T7 cell line and the kidney cells from a different monkey (AGMK4). Included in Fig 6 were the cells from the tumors and lung metastases formed by the AGMK1-9T7 p40 cells in nude mice. Both these assays demonstrated that GLI1 was expressed by all of these cells.

### Differentiation assays for adipocytes, osteoblasts, chondrocytes

**Fig 8:**
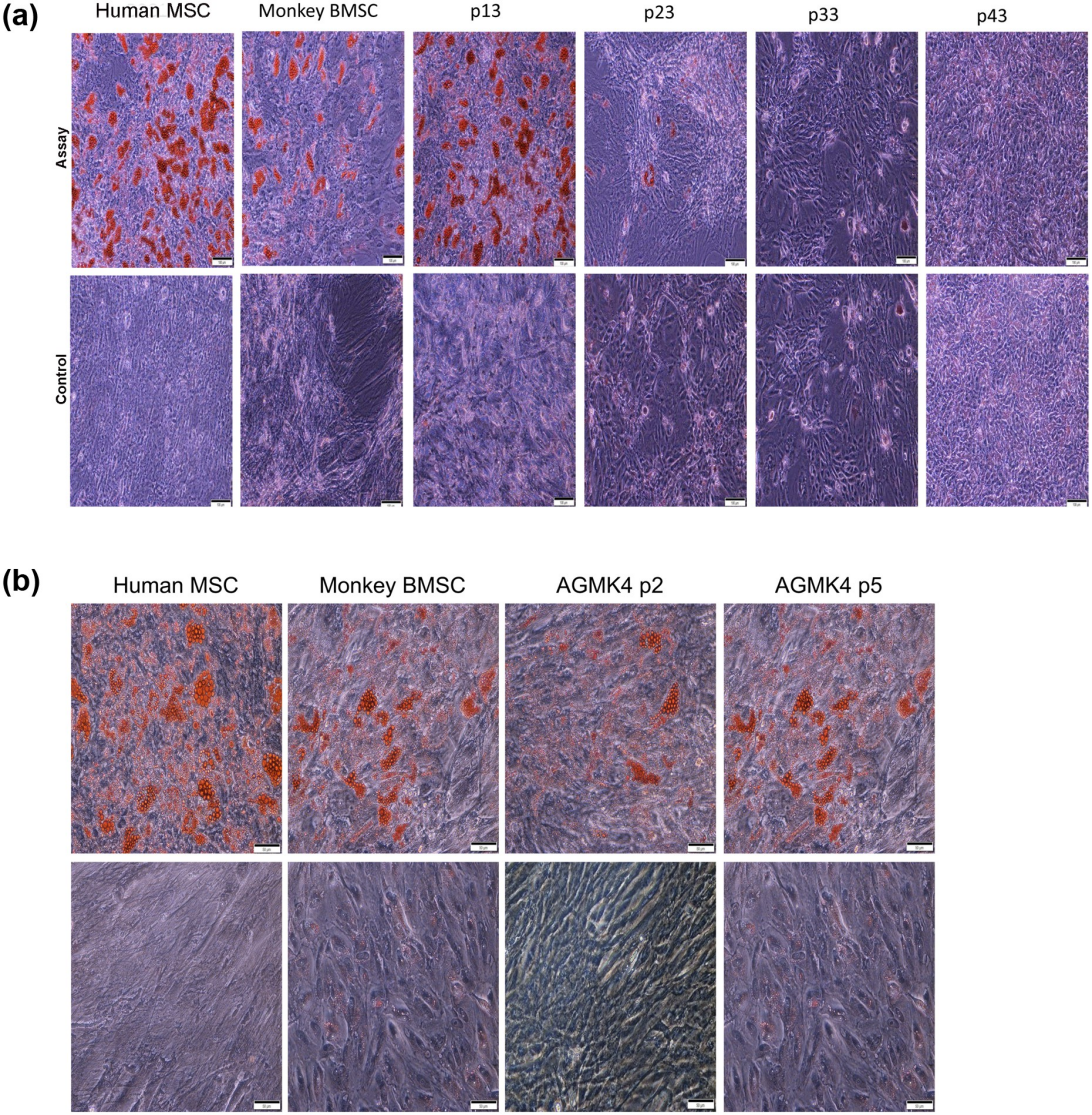
**a** Adipocytic differentiation assay with cells from the AGMK1-9T7 cell line. The cells that differentiated to form adipocytes stained bright red. Human MSC = human mesenchymal stromal cells [30]. The magnification for pictures in 8a and 8b is 4X. The Human MSC and Monkey BMSC (monkey bone marrow stem cells) are the control for these assays. **b**. Adipocytic differentiation assays using AGMK4 cells at p2 and p5. The cells from this monkey appeared to express fewer adipocytic cells at p2 compared with p5. The Human MSC and Monkey BMSC are the controls for these assays.

**Fig 9:**
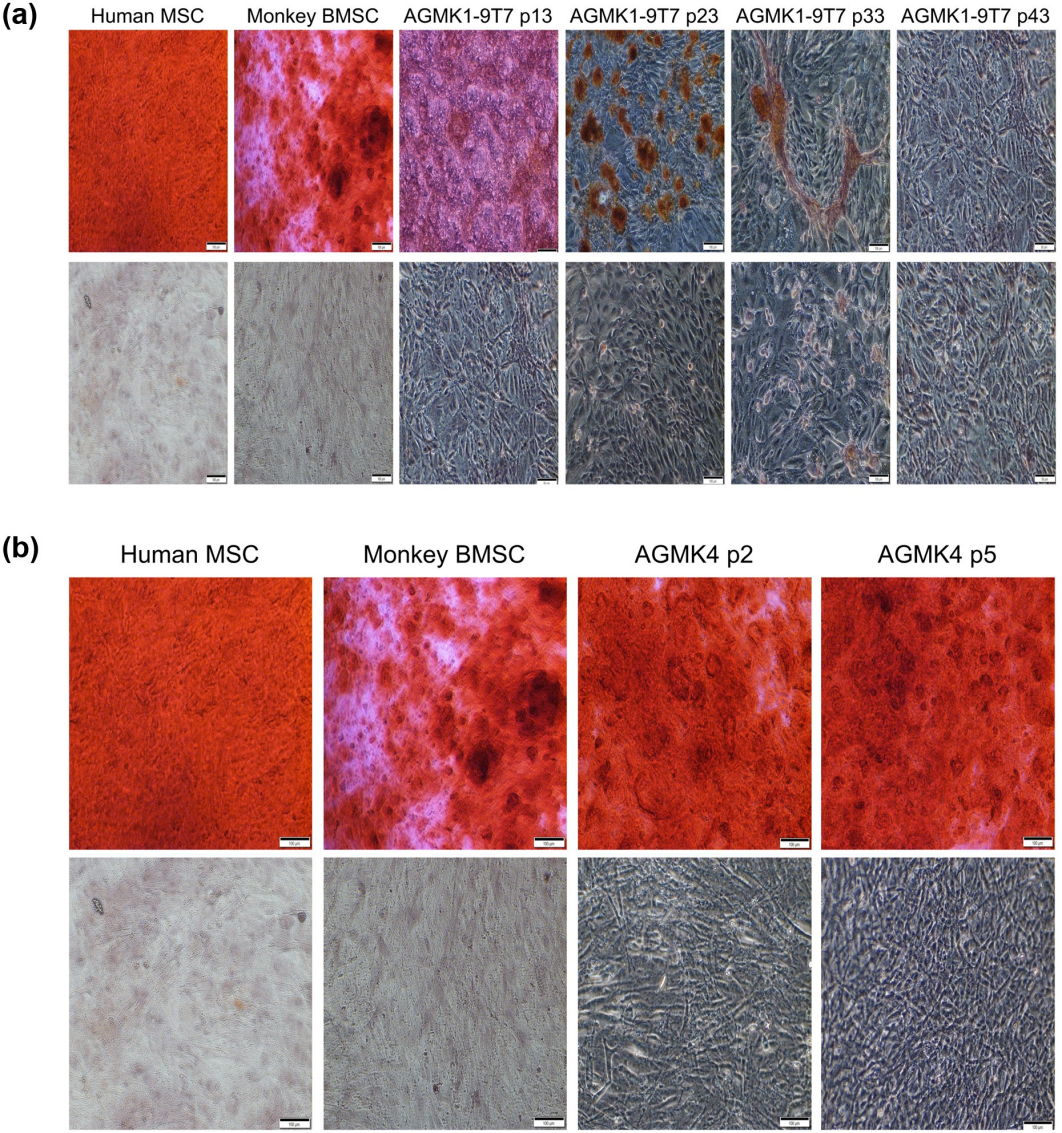
**a**. Osteogenic differentiation assays with AGMK1-9T7 cells at p13, p23, p33, p43. The osteocytes in this assay stained dark orange red. Human MSC = human mesenchymal stromal cells [30]. The magnification for pictures in 9a and 9b is 4X. The Human MSC and Monkey BMSC are the control for these assays. **b**. Osteogenic differentiation assays with AGMK cells at p2 and p5 from monkey AGMK4. Human MSC = human mesenchymal stromal cells [30]. The Human MSC and Monkey BMSC are the control for these assays.

**Fig 10:**
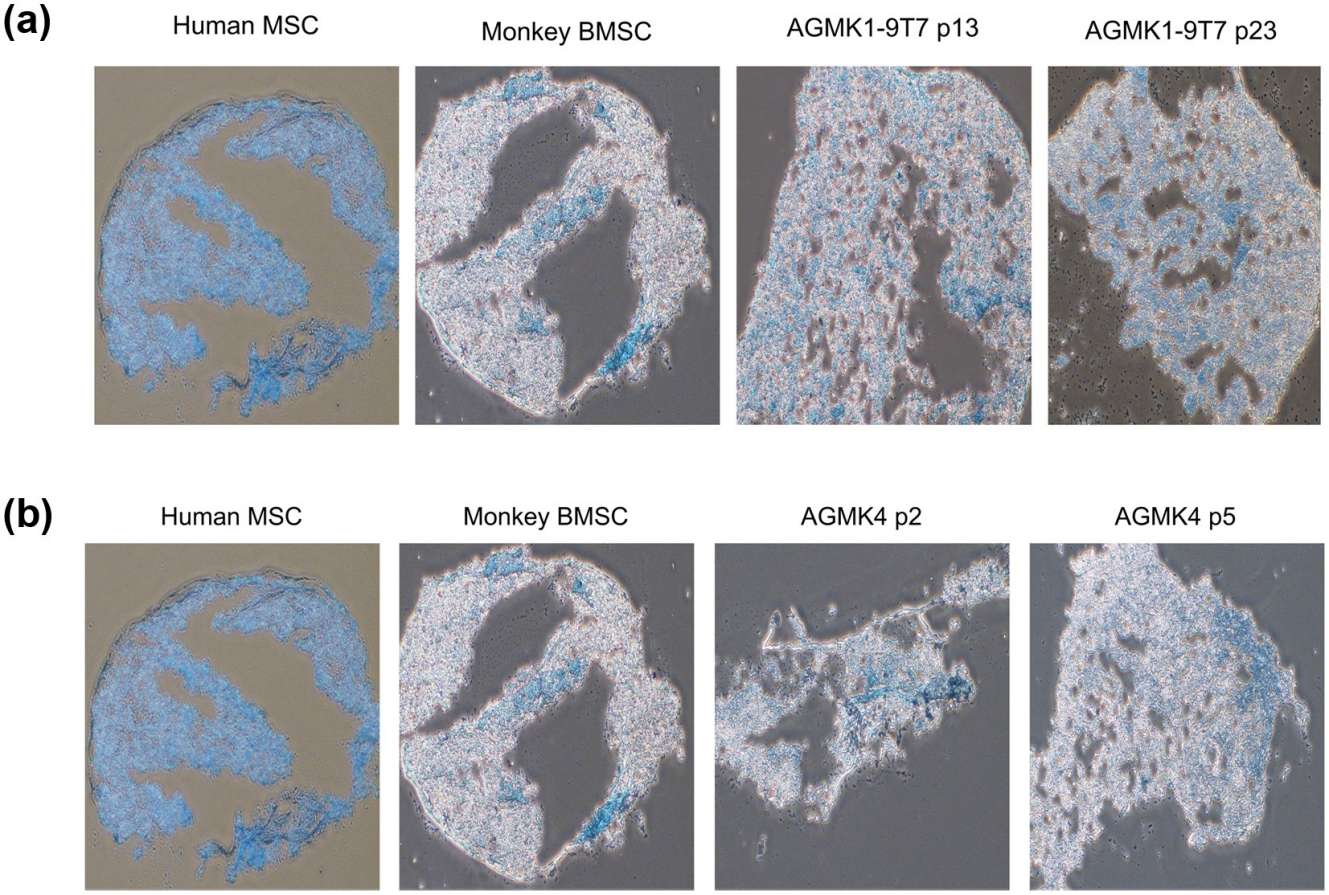
**a**. Chondrogenic differentiation assays with AGMK1-9T7 cells at p13 and p23. The chondroblasts in this assay stained bright blue. Human MSC = human mesenchymal stromal cells [30]. The magnification for pictures in 10a and 10b is 4X. The Human MSC and Monkey BMSC are the control for these assays. **b**. Chondrogenic differentiation assays with monkey AGMK4 cells at p2 and p5. Human MSC = human mesenchymal stromal cells. The Human MSC and Monkey BMSC are the control for these assays.

To continue the characterization of the AGMK1-9T7 cell line, and because these cells expressed markers that were indicative of being some type of stem cell or stem-cell progeny, we evaluated the ability of these cells to differentiate into adipocytes, osteoblasts, and chondrocytes; included in each of these assays were the p2 and p5 cells from the renal-cell suspension from another monkey (AGMK4). Adipocyte differentiation is shown in Fig 8a and 8b. Osteogenic differentiation is shown in Fig 9a and 9b. And chondrocyte differentiation is shown in Fig 10a and 10b. Human mesenchymal stromal cells (HMSC) [30] and commercially obtained rhesus monkey bone marrow stem cells (BMSC) were used as positive controls. AGMK4 at p5 and AGMK1-9T7 cells at passages p13 and p23 exhibited the capacity to differentiate into these 3 lineages. Interestingly, once the AGMK1-9T7 cells had been carried to p33 or higher passages, they lost their capacity to undergo differentiation. With respect to chondrocyte differentiation, when placed in chondrocyte-differentiating medium, the AGMK1-9T7 cells at p33 failed to form the pellets that formed at p5, p13, and p23. We are not aware of reports describing this passage-dependent capacity for differentiation, but it is consistent with the failure of these cells after p23 to differentiate into adipocytes and osteoblasts. These results suggest that the cells from AGMK1 that generated the AGMK1-9T7 cell line were some type of tissue-mature, adult renal cells that evolved to express a tumorigenic phenotype. During this evolution to become cells that can form tumors in vivo, they lost their ability to differentiate in these differentiation assays.

## Discussion

Based on the data described in this report, we have used the term progenitor cells to describe the cells from African green monkey kidney 1 that grew out in culture to form the AGMK1-9T7 cell line and that evolved over 40 passages to become tumorigenic and metastatic [8]. Progenitor cells have been defined as the descendants of stem cells that are involved in tissue repair and are capable of differentiating into cells that belong to the tissue/organ in which they reside (Wikipedia) [10]. The data presented in this report indicate that the AGMK1-9T7 cells are evolving mesenchymal progenitor cells that possess the capacity to become immortal fibroblasts that can differentiate to become adipocytes, osteoblasts, and chondrocytes and can evolve by additional passaging to express a tumorigenic phenotype. For this report, we are using the term progenitor cells to describe the cells that formed the AGMK1-9T7 cell line.

It has been suggested that pericytes as mesenchymal cells may play a role in the regeneration of some organs following injury [31–33]. It is possible, therefore, that pericytes may have produced some of the GLI1+ progenitor cells that formed the AGMK1-9T7 cell line. However, there are other reports that pericytes do not behave as mesenchymal/progenitor cells to create organ repair [34].

Progenitor cells do not have the ability to divide and reproduce indefinitely. However, mutations have been reported to occur in hematopoietic progenitor cells that convey self-renewal properties to these cells that allow them to become the origin of hematopoietic malignancies [35, 36]; these studies also described the possible role of mutations in the evolution of progenitor cells as they become more prolific. Based on these data, it appears that the progenitor cells that evolved to become the AGMK1-9T7 cells and that became tumorigenic at p40 likely represent the progeny of the progenitor cells that grew out in culture to form the AGMK1-9T7 cell line. So far as we are aware, there have been no reports describing the neoplastic initiation of progenitor cells from the organs of mammalian species. In our description of the AGMK1-9T7 cell line model of neoplastic development, the cells that grew out to form this cell line appear to have become neoplastically initiated at p1 [5].

Since the original description by Gey [2] and Earle and Nettleship [1] of the fibroblasts of the rat and mouse cells that evolved during cell-culture passage to become tumorigenic, other studies have described the fibroblastic morphology of a number of tissue-culture-derived cells that evolved in vitro to express a tumorigenic phenotype [18–22]. As described by Kramann et al. [10], our studies appear to have identified the AGMK cells that survive in culture and produce progeny and that evolve during neoplastic development (SPNDT) to be GLI1+ perivascular, renal, mesenchymal, progenitor cells that have developed due to tissue injury to become mesenchymal/fibroblastic in morphology [8]. This morphology persisted until p36, when these cells began to undergo mesenchymal to epithelial transition (MET) and became a line of epithelial cells by p40. These AGMK cells at p4 expressed GLI1, as detected by flow cytometry, western blot, and indirect immunofluorescence; the expression of this protein persisted through passages to p40 (Figs 5a, 6 and 7). These data support the hypothesis that the cells that formed the AGMK1-9T7 cell line are the progeny of perivascular GLI1+ renal, mesenchymal, progenitor cells present in the AGMK kidney.

These data suggest that the process of neoplasia in tissue culture represented by the AGMK1-9T7 cells could be a consequence of acquired mutations or due to epigenetic changes leading to de-differentiation of the tissue-mature, adult, renal progenitor cells present in this tissue, and that these cells possessed the ability to grow and produce progeny under these cell-culture conditions. The expression of their tumorigenic phenotype likely is an outcome of these mutations or epigenetic changes. These data have allowed us to develop a hypothetical model of neoplasia in vitro. “Normal” cells that grow in cell culture represent progenitor cell progeny. Perivascular, GLI1+ renal, mesenchymal, progenitor cells appear to be the cells that produced the AGMK1-9T7 cell line and likely also the VERO, BSC-1, and CV-1 AGMK cell lines, which all developed as fibroblasts. These results are supported by the ability of most mammalian cells to evolve to become immortal and tumorigenic. Human cells seem to be an exception to this, as there are not many examples of primary human cells becoming immortal by passaging in cell culture [37, 38]. Furthermore, these data can be interpreted to imply that the mammalian cells that survive and grow on plastic being sustained by artificial cell-culture medium containing serum from other mammals represent progenitor cell progeny as well. Based on these interpretations, “normal” cell components of mammalian tissues that are not some type of progenitor-cell progeny would not have the ability to survive when placed in a tissue-culture environment. This hypothesis is supported by data from the in vivo models of tumorigenicity, which have shown that neoplastically initiated cells can evolve along multiple pathways interspersed from “ultimate malignancy to extinction (apoptosis), or to 4 intermediate stages” [39] of neoplasia during their growth in animals [4, 40]. These data appear to be supported by our study of AGMK cells growing in cell culture.

Our studies on the AGMK1-9T7 cell line have allowed us to identify the likely source of the cells that evolve from normal cells to tumor cells in this non-human primate cell model. The source of these cells appears to be renal, perivascular, mesenchymal, progenitor cells that express GLI1, a protein that has been shown to be expressed by mesenchymal progenitor cells that become unipotent in severely injured kidneys and provide the fibroblasts to heal these extensive injuries [10]. When suspensions of renal cells from disrupted monkey kidneys are plated in culture, the cells that grow to form cell-culture monolayers appear to be the progeny of these GLI1+ fibroblasts. During the process of evolving to form cell monolayers, these GLI1+ fibroblasts appear to become neoplastically initiated [5] and begin their neoplastic evolution toward expressing a tumorigenic phenotype. The identification of these GLI1-expressing fibroblasts as the source of neoplasia in vitro might represent one of the fundamentals of this biological process. It might suggest that most, possibly all, of the cells grown in culture from the disintegrated organs used to produce such cells could evolve by similar processes. This process could be the origin of tumor cells both in vitro and in vivo. Defining the neoplastic events that occur across the spectrum of neoplasia provides a systematic method for evaluating and understanding the molecular mechanisms of neoplasia in cell culture.

## Supporting information

S1 Data miRNAs expressed in the kidneys of 5 different African green monkeys. (XLSX)

S2 Data miRNAs expressed at P1 and their association with cancers in humans. (XLSX)

S3 Data Original gel used to develop Fig 7. (DOCX)

## Abbreviations

AGMK: African green monkey kidney
miRNA: MicroRNA
MSC: mesenchymal stromal cells
SPNDT: spontaneous neoplastic development/transformation
