# Supplementary material for: GLI1+ perivascular, renal, progenitor cells: The likely source of spontaneous neoplasia that created the AGMK1-9T7 cell line
Source: PLoS One. 2023 Dec 7;18(12):e0293406. doi: 10.1371/journal.pone.0293406 (PMC10703308; doi:10.1371/journal.pone.0293406)

# Supporting Data File 3: Reaction observed by staining for GLI1 and β Actin

# Original:


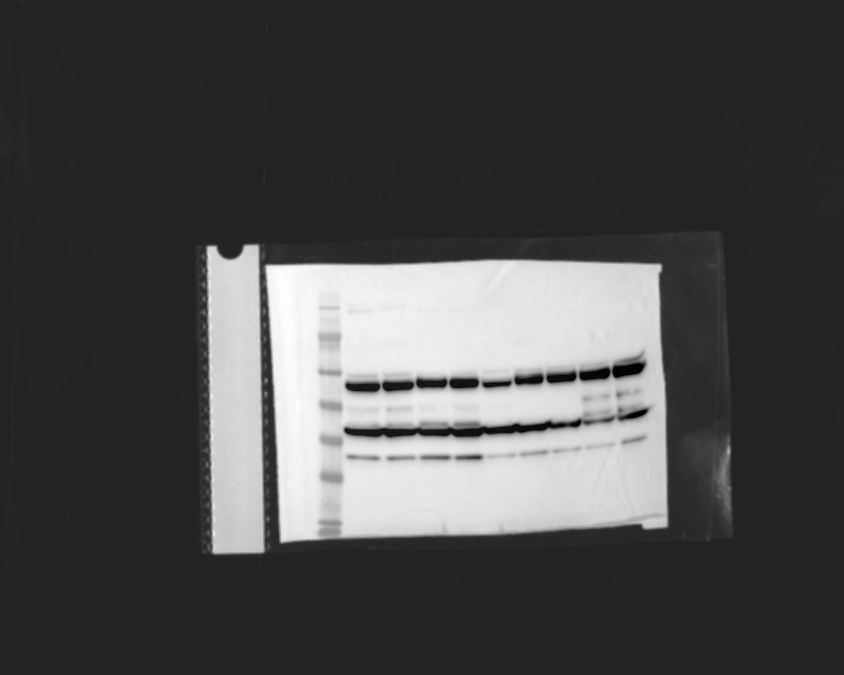


As presented in paper:


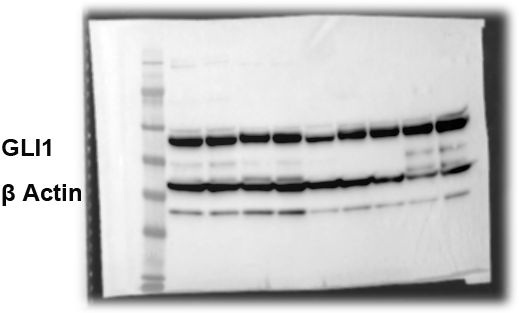

Supplement: S3 Data — (DOCX) [file pone.0293406.s003.docx]
